# Good Accounting and the Uniform System

**Published:** 1911-10-14

**Authors:** 


### HOSPITAL ECONOMICS.

(Contributions to this Section are Invited.)

### Good Accounting and the Uniform System.

It has always been a matter of controversy as to how far hospital statistics can be relied upon. All reliable financial statistics must rest upon good accounting. It is desirable therefore to introduce this section by tracing the origin of the uniform system of institutional accounts. Forty years ago there was no such thing as uniformity in institutional accounts, and every institution kept such books or apologies for books as the ability and zeal or the relative indifference and ignorance of those responsible set up in connection with each individual charity. It was not so much the want of zeal as a want of knowledge which caused the evil.

In 1868 the Queen's Hospital, Birmingham, after a chequered career full of vicissitudes and strange adventures—we are speaking of the days when the late Sands Cox was a power in Birmingham—decided to make a supreme effort to put its house in order. Much of the credit of the important changes which resulted is due to Mr. Alderman Johnson, who was then solicitor to the Queen's Hospital, and who was as untiring and generous in his efforts for improvement as his great abilities and knowledge of business were invaluable to the success which resulted from these efforts. At that time the accounts, such as they were, were kept on a system of single entry and the results left much to desire. The late Mr. Laundy, a famous accountant of the Midlands, was the auditor, and co-operated with the new secretary in 1868 in laying down a system of accounts which would show every item of expenditure under its proper head and enable the managers from week to week, and month to month, throughout each year to compare the outgoings under every head and to keep a close control over the expenditure in every detail. The new system of accounts was also planned so as to give clearly and distinctly every main source of income, and to show whether it was progressively increasing or falling off. It soon became apparent that the new system of accounts was invaluable, for it produced not only reduction in expenditure but a steady growth in income and a continuous development of the sources from which this income was derived. One fact which became speedily apparent was that the artisans of Birmingham did not contribute regularly or in any real sense to the support of the hospitals.

The late Mr. Sampson Gamgee, one of the Honorary Surgeons to the Queen's Hospital, who was a well-known Birmingham worthy in his day, obtained the sanction of the committee to call together the leading representatives of the working classes with a view to secure their interest in the work of the hospitals and to promote an organised effort to obtain subscriptions from the workshops. It was resolved to ask the working men of Birmingham to contribute £5,000 to the Queen's Hospital Extension Fund. The movement met with considerable opposition in certain quarters, but it won the sympathies of the artisans of Birmingham to a surprising extent and resulted in the collection within three years of £4,058. This money was devoted to the erection of an out-patient department on original lines, which proved so successful that this new unit in hospital construction has practically ever since constituted the basis for all the best constructed out-patient departments not only in Great Britain but in the United States of America too. We have ventured to call to recollection these facts because the first fruits of an adequate system of hospital accounts led initially to the first great effort successfully to win the monetary support of the working classes for the voluntary hospitals and eventuated in the Hospital Saturday movement.

It must be borne in mind that the developments for improving the administration of the Queen's Hospitals soon spread abroad, and the more active spirits in the hospital world began to visit it with the view to understanding their nature and value. In fact, as we hope to show one day, the beginning of the modern system of training nurses on practical lines had its origin at the Queen's Hospital, Birmingham. The new system of accounts and the clear way in which everything was presented to the Governors in the Annual Reports had of course a special interest for hospital workers in the seventies, and they gradually came to adopt an identical system, and so the system spread, continuously. When the late Sir Sydney Waterlow was Lord Mayor of London, the secretary of the Queen's Hospital was asked to prepare the necessary papers with a scheme for introducing Hospital Sunday into the Metropolis as Sir Sydney Waterlow wished to do. When the secretary was appointed to take charge of the Dreadnought Seamen's Hospital, he was soon invited to become a member of the Council of the Metropolitan Hospital Sunday Fund, having meanwhile developed the system of accounts already referred to and used his efforts to get it generally adopted as the uniform system of hospital accounts. In a few years the Metropolitan Hospital Sunday Fund made a rule that all institutions seeking grants from this fund must keep their accounts on the uniform system. One important development had taken place, the addition of a classified index of items under all heads which were included in the uniform system. The King's Fund adopted the same course, and with the aid of committees of hospital secretaries the two funds have made this classified index to-day as comprehensive and complete as possible. It will thus be seen that the uniform system of accounts has been carefully elaborated and perfected until it forms to-day the most reliable basis extant for hospital statistics, not only in the United Kingdom but throughout the Empire, many of the Colonies having adopted it.

